# Supplementary material for: Chronic Heat Stress Affects Bile Acid Profile and Gut Microbiota in Broilers
Source: Int J Mol Sci. 2023 Jun 16;24(12):10238. doi: 10.3390/ijms241210238 (PMC10299590; doi:10.3390/ijms241210238)
Supplement: Supplementary file 1 [file ijms-24-10238-s001.zip › ijms-2433637-supplementary.pdf]

Table S1. Real-time PCR primer sequences.

Target genes	Primer sequences (5′ to 3′)
CYP27A1	F: ACTTTCGTCTGGCTCTTCCTG<br>R: CATCGGGTATTTGCCCTCCT
CYP7A1	F: TGCTCCGCATGTTCCTGAAT<br>R: AGAAGGTAAACAAGCTCCAAAAAGT
CYP7B1	F: CATGTGTCCAGGGAGGTTCC<br>R: TGTCTGGCAGGATACCA
CYP8B1	F: CCTTTCGGAGACGAAGACCC<br>R: AAGTCCAGTGCGTAACCCAG
BSEP	F: TGGAATAGAGCGTGGCTTTT<br>R: CATTGGCAGTCATCTCAGGA
MRP2	F: TCTGCTTGTGCAGAGACTCG<br>R: TACATCCACGATGGGGTCCT
NTCP	F: TCAAGGGAGCCTCAAAGAGC<br>R: CTGAGTGCAAATGGTGGTGC
OATP1B3	F: TCTGGCTGGATGCACGATTT<br>R: TCAACTCTGGCTGAACGCAT
ASBT	F: GGGGATGATGCCACTCTGTC<br>R: CCCCCAACCACAGCAGTAAT
OSTα	F: GCTGGACATGGTCCAACTCA<br>R: CACCATCATGGAACGTGGGA
OSTβ	F: AAGTCTGCATGCATGATTGGC<br>R: CAAAGGCACGTTCCACATCG
FXR	F: AGTAGAAGCCATGTTCCTCCGTT<br>R: GCAGTGCATATTCCTCCTGTGTC
SHP	F: TTCCAAATGCGGGGCCTATT<br>R: TCAATGTCACAGTCCAGCCC
FGF19	F: CCGCAGAGTCTGTTGGAGAT<br>R: GTTGTAGCCGTCTGGACGAA

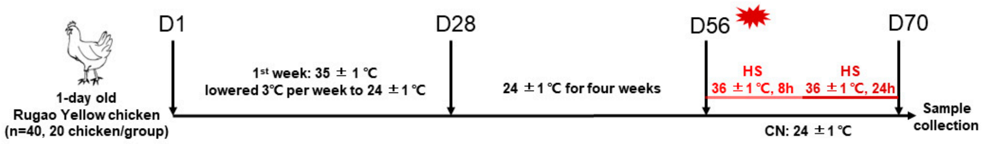

Figure S1. Animals and Experimental Design.
